# Nosocomial tuberculosis transmission from 2006 to 2018 in Beijing Chest Hospital, China

**DOI:** 10.1186/s13756-020-00831-5

**Published:** 2020-10-24

**Authors:** Zhongyao Xie, Ning Zhou, Yuqing Chi, Guofang Huang, Jingping Wang, Hui Gao, Na Xie, Qianhui Ma, Nan Yang, Zhenlan Duan, Wenjuan Nie, Zhaogang Sun, Naihui Chu

**Affiliations:** 1 Nosocomial Infections Control Department, Beijing Chest Hospital Affiliated to Capital Medical University, No 9, Beiguan Street, Tongzhou District, Beijing, 101149 People’s Republic of China; 2 Beijing Chest Hospital Affiliated to Capital Medical University, No 9, Beiguan Street, Tongzhou District, Beijing, 101149 People’s Republic of China; 3 Human Resources Department, Beijing Chest Hospital Affiliated to Capital Medical University, No 9, Beiguan Street, Tongzhou District, Beijing, 101149 People’s Republic of China; 4 Tuberculosis Department, Beijing Chest Hospital Affiliated to Capital Medical University, No 9, Beiguan Street, Tongzhou District, Beijing, 101149 People’s Republic of China

**Keywords:** Nosocomial transmission, Tuberculosis, Respirator, Ultraviolet, Ventilation

## Abstract

**Introduction:**

Strong evidence is lacking to support effectiveness of currently implemented tuberculosis infection prevention control (TB-IPC) measures for preventing nosocomial tuberculosis (TB) transmission. This 13-year analysis is the longest follow-up investigation to date to identify risk factors underlying nosocomial TB transmission.

**Methods:**

We monitored all staff of Beijing Chest Hospital each year from 2006 to 2018. Age, gender, duration, department, education, income, respirator, ultraviolet, and ventilation were chosen as variables. Univariate cox regression, correlation analysis, and multivariate cox regression were analyzed sequentially.

**Results:**

Using multivariable cox regression analysis, variables of income, ultraviolet germicidal irradiation (UVGI), natural ventilation and mechanical ventilation conferred significant protective effects, with odds ratios of 0.499, 0.058, 0.003, and 0.015, respectively (*P* < 0.05). Medical N95 respirator conferred an excellent protective effect, with an associated TB infection rate of 0%. Notably, inadequately maintained mechanical ventilation systems were less protective than natural ventilation systems.

**Conclusion:**

UVGI, adequate ventilation, and use of medical N95 respirator may be risk factors of nosocomial TB transmission.

## Introduction

Nosocomial tuberculosis (TB) transmission among health care workers is a well-documented occupational risk factor [1–4]. Indeed, TB transmission risk among patients and health care workers well recognized [5–7], leading to greater TB incidence rates for health care workers than for the general population [8–13]. In fact, in regions where specialized TB hospitals are found, the risk for TB infection among hospital staff can reach 7.5–60 times that of the general population [14, 15].

There has been renewed interest in TB infection control, especially in areas with high TB and HIV prevalence [16, 17]. Consequently, several TB infection prevention control (TB-IPC) measures have been proposed, although most are only realistically feasible in high-income countries [18], not in low-income settings [19]. Meanwhile, other TB-IPC recommendations appropriate to any setting lack strong evidence of effectiveness [20]. Therefore, analysis of risk factors for nosocomial TB transmission is needed to guide best TB-IBC practices going forward, especially regarding use of expensive and cumbersome protective equipment, as recommended for controlling epidemic outbreaks (e.g., Covid-19 coronavirus).

Several previous studies have compared various types of masks or respirators for protective effectiveness against clinical respiratory illnesses. N95 which costs higher price was thought as the more advanced protective equipment. But recent study among nurses in hospitals, use of a surgical mask (medical mask) compared with an N95 respirator resulted in noninferior rates of laboratory-confirmed influenza […]. However, those studies did not specifically focus on TB infection, were largely cross-sectional, lacked long-term follow-up [21], and lacked strong evidence of efficacy. Therefore, this study was conducted as a first cohort study following all staff of Beijing Chest Hospital for a period of 13 years with the goal to determine risk factors underlying nosocomial TB transmission.

## Method

Beijing Chest Hospital is a TB diagnostic and treatment center in China. All hospital staff are at risk of exposure to TB patients and/or contaminated materials in spite of hospital utilization of air-cleaning equipment, including recommended ventilation systems and/or ultraviolet lamps. Therefore, as an added safety precaution all workers handling contaminated materials or in contact with TB patients are advised to wear masks.

This retrospective cohort study used 13 years of data (2006–2018) from Network Query System of Beijing Chest Hospital (NQS), a database maintained by Beijing Chest Hospital affiliated to Capital Medical University. In 2019 we started to collect demographic data included age, sex, duration (years of working), education, income, respirator, ultraviolet and ventilation. From 2006 to 2018, physical examinations of hospital staff were performed every year. Case diagnosis was based on clinical signs and radiological, serological, and bacteriological investigations. Nosocomial TB transmission was detected by a change in infection status from negative to positive, as detected using purified protein derivative (PPD) skin tests or interferon-gamma release assays (IGRAs) with or without positive sputum test results or detection of pulmonary TB lesions via computer radiography (CR) or computed tomography (CT). Data including.

To identify potential risk factors of nosocomial TB transmission, a two-step process was implemented as follows: (1) Summarize previous study results; (2) Discuss results with clinical researchers and epidemiologists. Next, we selected age, sex, duration (years of working), department, education, income, respirator, ultraviolet, and ventilation as variables for analysis. An electronic database was maintained to store individual demographic, clinical, and epidemiological data and results of physical examinations and serological and radiological testing collected from 2006 to 2018.

Statistical analysis was conducted using SPSS software (version 24). We used univariate cox regression to evaluate variables of age, sex, duration, department, education, income, respirator, ultraviolet, and ventilation as the potential risk factors. Age and duration were classified as continuous variables, while other variables were classified as categorical variables. Significance was set at *P* < 0.1 using two-sided comparisons. Variables above with *P* < 0.1 were chosen for analysis via the bivariate correlation test, while variables with low correlation coefficients were selected for analysis in the next step using multivariate cox regression. Correlations between continuous variables were evaluated using two-tailed Pearson test and for categorical variables using two-tailed Spearman test. Variables with *P* < 0.01 and correlation values > 0.5 were deemed significant correlations, while only one variable could be selected for subsequent multivariate cox regression analysis. For multivariate cox regression analysis, the first sub-variable was chosen as the reference category then backward and forward selection methods were used to identify independent predictors. Factors with *P* < 0.05 in multivariate cox regression analysis were considered risk factors with significant impacts on TB infection risk.

## Result

Additional file 1: Fig. S1 shows TB infection rates every year from 2006 to 2018. Notably, in spite of changing TB-IPC measures over time, masks were used as personal protective equipment for most of the study period. However, prior to 2012, staff wore gauze masks when at risk of TB exposure. From 2013 to 2015, medical masks replaced gauze masks in most department but still some department use gauze mask, and in 2016 medical N95 respirators replaced medical masks in most department but also some department still use gauze mask for its reusability (Additional file 1: Fig. S1). Although ultraviolet irradiation and ventilation were used for environmental TB control prior to 2009, ultraviolet germicidal irradiation (UVGI) treatment during that time was only used for bed sterilization after patient discharge; since 2010, UVGI fixtures have been used daily. Regarding ventilation, prior to 2010 ventilation was based on natural air exchange, while in 2011 natural ventilation systems were replaced with mechanical ventilation systems. In Additional file 1: Fig. S1, although natural ventilation was replaced by mechanical after 2011, mechanical ventilation was mainly used in department which deeply exposed to TB, other departments with slight exposure still used natural ventilation.

Each year new employees joined in the hospital staff and older employees retired. The sample size from 2006 to 2018 was 977, 941, 945, 953, 941, 943, 965, 943, 941, 941, 947, 974 and 976, respectively. The baseline of TST /IGRA positive was 20 in 2018, and these 20 cases was excluded in the next period. The HCW who had TST /IGRA conversion from 2006 to 2018 was 20, 4, 8, 2, 3, 14, 3, 2, 2, 5, 1 and 2, respectively. For the timely detection of TB infection by CT, none of these HCW who had TST /IGRA conversion developed to sputum-positive TB patients. Table 1 shows staff and TB-IPC characteristics in 2018, with distribution of variables for each category (age, sex, duration, education, income, respirator, ultraviolet, and ventilation) and staff proportions as shown. Workers who left prior to 2018 included retired workers (reaching retirement age) and resigned workers (because of family or personal reason). For the ones leaving their position only cover a little percent of the whole staff in our hospital, the mean value of each variable in each year doesn’t vary. In Table 1, 69% of staff are undergraduate, including nurses, housekeeping, management staff, clerical workers, maintenance workers, security personnel, ancillary workers, some radiology technicians, pharmacists and laboratory workers.

**Table 1:** Characteristics of all the staff and different TB-IPC measures in Beijing Chest Hospital in 2018

Variable	Mean or number (%) (n = 977)
Age	40
Sex	
Male	282 (28.9%)
Duration of working time	16 (1.6%)
Education	
Less than undergraduate	75 (7.7%)
Undergraduate	675 (69.1%)
Graduate and postgraduate	226 (23.1%)
Income	
Equal and under average	611 (62.5%)
Above average	365 (37.4%)
Respirator	
No respirator	584 (60.1%)
Gauze mask	177 (18.1%)
Medical mask	0 (0%)
High-level N95 respirator	215 (22.0%)
Ultraviolet	
No ultraviolet	215 (22.0%)
Ultraviolet only for bed	0 (0%)
Air UVGI	761 (77.9%)
Ventilation	
Natural ventilation	733 (75.0%)
Mechanical ventilation	243 (24.9%)

According to the aforementioned changes in TB-IPC measures, the period from 2006 to 2018 could be divided into early TB- IPC (2006–2010), intermediate TB-IPC (2011–2014), and enhanced TB-IPC (2015–2018) time periods (Table 2). Before 2010 (early TB-IPC period), TB-IPC measures included gauze masks, natural ventilation, and UV treatment only of beds. From 2011 to 2014 (intermediate TB-IPC period), medical masks, mechanical ventilation, and UVGI were used. After 2015 (enhanced TB-IPC period), medical masks were replaced with medical N95 respirators. Average TB infection rates per year for each TB-IPC period are shown in Table 2. In the enhanced TB-IPC period, a significant reduction in TB infection incidence rate was observed relative to incidence during the early TB-IPC period, when only limited TB-IPC measures were available.

**Table 2:** The TB infection rate in different period was compared by SPSS software. The period from 2006 to 2018 could be divided into early TB-IPC (2006–2010) and enhanced TB-IPC (2015–2018) according to the change of TB-IPC measure. The early TB-IPC (2006–2010) was set for reference group

	The average TB incidence rate per year in TB exposed environment (‰)	*P* value
Early TB-IPC (2006–2010)	32.1	
Intermediate TB-IPC (2011–2014)	24.4	0.350
Enhanced TB-IPC (2015–2018)	11.3	0.002

The prevalence of TB infection was analyzed against variables of age, gender, duration, workers’ education, income, respirator, ultraviolet, and ventilation, with univariate cox regression results presented in Table 3. Age, duration**,** department, income, respirator, ultraviolet, and ventilation were selected as potential predictors of nosocomial TB transmission (*P* < 0.1).

**Table 3:** Factors associated with TB infection incidence analyzed by univariate cox regression analysis

Variable	*P* value	OR	95% CI for Exp(B)	
			Lower	Upper
Age	0.010	0.969	0.945	0.992
Sex				
Male	0.127		0.352	1.139
Female				
Duration	0.091		0.959	1.003
Education				
Less than undergraduate	0.784			
Undergraduate	0.668		0.299	2.168
Graduate and postgraduate	0.500		0.480	1.431
Income				
Equal and less than average	0.005	0.429	0.238	0.772
Above average				
Respirator				
No respirator	0.000			
Gauze mask	0.000	3.725	2.229	6.226
Medical mask	0.067	2.329	0.942	5.757
Medical N95 respirator	0.950	0.000	0.000	3.898E+155
Ultraviolet				
No ultraviolet	0.000			
Ultraviolet only for bed	0.000	4.524	2.080	9.839
Air UVGI	0.000	0.232	0.136	0.397
Ventilation				
No ventilation	0.000			
Natural ventilation	0.000	0.229	0.130	0.403
Mechanical ventilation	0.031	0.526	0.293	0.944

Bivariate correlations were analyzed using Pearson or Spearman tests (Additional file 1: Table S1). Age and duration were highly correlated, with a Pearson correlation value of 0.886 (*P* = 0.000). To control the interaction between these two variables, only duration was chosen for the next multivariable analysis. Correlations between ultraviolet and respirator and between ventilation and respirator were low (0.361 and 0.408, respectively, *P* = 0.000). As ultraviolet and ventilation showed a mid-level correlation (Spearman correlation value of 0.539, *P* = 0.000), they were both selected for multivariable cox regression. No associations were found between other pairs of variables.

Next, multivariable cox regression analyses including duration, income, respirator, ultraviolet, and ventilation were performed (Table 4). Although a significant predictor in univariable cox regression, duration showed no significant influence on TB infection (*P* > 0.05), while income was a protective predictor (*P* < 0.05), with an OR value of 0.499 (95% CI 0.273–0.915). Respirator was stratified into no respirator, gauze mask, medical mask, and medical N95 respirator, with no respirator serving as reference category to yield OR values for gauze mask, medical mask, and medical N95 respirator of 233.093 (*P* = 0.000), 46.580 (*P* = 0.000), and 0.000 (*P* = 0.969), respectively. Notably, these results indicate that use of gauze masks and medical masks are risk factors for TB infection, while a result of *P* > 0.05 obtained for medical N95 respirator indicated an insignificant correlation with risk. Nevertheless, use of a medical N95 respirator conferred excellent protection, with 0% TB infection observed for those using this device. Meanwhile, using no ultraviolet irradiation as the reference category, use of an air UVGI system protected against TB infection, with an OR value of 0.058 (*P* = 0.000); using no ventilation as a reference variable, both natural ventilation and mechanical ventilation showed protective influences against TB infection (*P* = 0.05). However, comparison of protective effects of natural versus mechanical ventilation demonstrated that natural ventilation had a greater protective effect against TB infection (*P* = 0.000, OR 0.222). This result prompted hospital maintenance workers to test mechanical ventilation systems carefully to detect contamination with TB-causing bacteria. Subsequently, results of testing led to recommendations that more frequent replacement of filter screens be incorporated into routine maintenance procedures for these systems.

**Table 4:** Multivariable statistics of TB infection incidence survival by cox regression analysis

Variable	Sub-variable	B	P value	OR	95% CI	
					Lower	Upper
Duration		− 0.022	0.061	0.978	0.955	1.001
Income	Equal and less than average	− 0.694	0.025	0.499	0.273	0.915
	Above average					
Respirator	No respirator		0.000			
	Gauze mask	5.451	0.000	233.093	80.583	674.237
	Medical mask	3.841	0.000	46.580	11.268	192.560
	Medical N95 respirator	− 9.533	0.969	0.000	0.000	2.521E+203
Ventilation	No ventilation		0.000			
	Natural ventilation	− 5.691	0.000	0.003	0.001	0.010
	Mechanical ventilation	− 4.186	0.000	0.015	0.005	0.046
Ultraviolet	No ultraviolet		0.000			
	Ultraviolet only for bed	− 0.227	0.588	0.797	0.351	1.810
	Air UVGI	− 2.853	0.000	0.058	0.026	0.127

## Discussion

Hospital staff members working in rooms occupied by TB patients were at significantly higher risk of developing active TB than were other employees [22]. To avoid hospital-based nosocomial TB infections, certain specific TB-IPC measures should be administered, while some previous recommended practices require justification due to weak or indirect evidence of effectiveness [20].

In our study, duration was not shown to be significantly correlated with TB nosocomial transmission (*P* = 0.061, OR 0.978, 95% CI 0.955–1.001), in opposition to results of previous studies showing that working less time in the hospital might lead to a lower rate of tuberculin skin test (TST) positivity [23]. To explain this discrepancy, here we postulated that elderly workers with long work experience and greater duration might implement practices that more effectively prevent them from contracting TB than practices used by new staff with less experience and lower accumulated duration.

Gender was previously thought to be a significant predictor of TB infection, as some studies indicated that men had higher TST positivity rates that were possibly due to a higher rate of community exposure among males [23]. By contrast, other studies have demonstrated that some specific female professional groups (nurses and laboratory technicians) had significantly higher rates of TST positivity, possibly reflecting the high proportion of females in the nursing field, a high-risk profession for TB infection. Nevertheless, in this study no significant correlation was observed between gender and nosocomial TB infection.

As previous studies have stressed the role of socioeconomic conditions in TB transmission among health care workers [24], here we evaluated income as a risk factor for TB infection and verified it to be a protective factor such that high-income correlated with lower TB incidence rate as compared to low-income status. In our hospital we note that high-income individuals may occupy more spacious working and living spaces and benefit from higher quality diets and healthier work and rest arrangements than those with low-income status, all of which may be relevant to TB prevention.

In theory, respirators may provide additional protection for health workers, who should use particulate respirators that prevent exposure to TB-causing mycobacteria released during high-risk aerosol-generating procedures that tend to confer a high risk of TB transmission (e.g. bronchoscopy, intubation, sputum induction procedures, aspiration of respiratory secretions, and autopsy or lung surgery using high-speed devices). In addition, respirator use is especially necessary to protect those providing care to infectious multidrug resistant TB (MDR-TB) and extensively drug resistant TB (XDR-TB) patients or people suspected of having infectious MDR-TB and XDR-TB. However, few studies to date have investigated whether use of particulate respirators is of value to those providing routine TB patient care in situations where administrative and environmental controls are in place [20]. In fact, only weak and indirect accumulated evidence supports the use of particulate respirators for TB infection control, prompting us to conduct this first study to compare mask and respirator use for protection against TB over an extended time period (13 years). Here we demonstrated that use of a medical N95 respirator conferred excellent protection against TB infection, with 0% TB incidence observed with use of this device. The reason for the lack of significance of this result may lie in the small number of cases studied (case number = 0). However, as a limitation of correct N95 respirator use, extensive conditions must be met: (1) requires training. (2) Requires adherence to best practices. (3) May adversely affect health worker’s performance during some procedures. (4) Reduces comfort of health workers. (5) No clear guidance exists on how long the same respirator can be used over time. Notably, an interesting observation of this study, that gauze and medical mask use might be a risk factor for TB infection, must be emphasized. This finding may reflect the fact that masks do not provide adequate protection and/or that workers wearing gauze and medical masks might engage in closer contact with infected patients or contaminated materials, with further investigation warranted to more fully understand mask-associated risk.

Priority should be given to achieving adequate air change per hour (ACH) values using ventilation systems, although this is not possible in some settings; for example, due to climatic conditions (e.g. in winter or during the night), in some building structures, or in situations where TB transmission would pose a high risk of morbidity and mortality (e.g. in MDR-TB wards). In such cases, a complementary measure incorporating use of upper-room or shielded UVGI devices may be an option, even though the effectiveness of such measures are only supported by weak evidence. For example, one epidemiologic study investigating TST conversion rates in health workers showed no major additional benefit; however, another well-designed animal model study (using guinea pigs) demonstrated that upper-room UVGI could reduce TB infection [20]. Here, air UVGI use was verified to be protective against TB infection, while ultraviolet use only for bed sterilization showed no significant protection. This discrepancy my reflect the fact that ultraviolet irradiation-based measures should be capable of achieving air disinfection equivalent to 10–20 ACH if the air UVGI system has been appropriately designed, installed, maintained, and operated. While ultraviolet irradiation measures are generally suitable for most climates, UVGI use has several weaknesses: (1) Requires expertise in design, installation and testing. (2) Requires maintenance and cleaning (not effective if not well maintained). (3) Requires air mixing to be effective, but no easy-to-use tool exists for measuring equivalent ACH. (4) Direct UVGI exposure or overexposure results in non-permanent adverse effects (photokeratitis and erythema), while upper UVGI devices are potentially hazardous if improperly designed or installed. In well-designed systems, the principal hazard is inadvertent eye exposure by workers climbing up into the high-UV zone to perform painting, cleaning, and maintenance tasks. As with any engineering control, use of a UVGI device requires proper design, installation, operation, and maintenance [20].

Importantly, UVGI devices do not replace ventilation systems; rather, they should be considered a complementary intervention. However, adequate ventilation in health-care facilities is essential for preventing transmission of airborne infections and is strongly recommended for controlling TB transmission, although such recommendations are based on low-quality indirect evidence lacking quantitative demonstration of impact of adequate ventilation on TB transmission. In our study, two ventilation systems were shown to be protective against TB infection, with mechanical ventilation less protective than natural ventilation. Consequently, the mechanical ventilation system used in our hospital was tested carefully and subsequent results indicated that the filters inside the system needed replacement. Thus, our result aligns with results of previous studies showing that health-care facilities relying on natural ventilation alone could maintain effective ventilation through proper system operation and regular maintenance. For example, simple natural ventilation may be optimized by maximizing the size of window openings and by locating windows on opposing walls; indeed, such systems are capable of achieving ACH above the required minimum of 12 ACH to ensure accelerated decay of droplet nuclei. However, natural ventilation has weaknesses: (1) Difficult to control (depends on wind and temperature). (2) No control over direction of air flow. (3) Sufficient permanent openings (e.g. windows and vents) should be guaranteed to maintain adequate ACH. (4) No easy-to-use tool for measuring ACH. (5) Limited applicability (only suitable in a few locations globally). Therefore, when natural ventilation alone cannot provide sufficient ventilation rates, well-designed, maintained, and operated fans (mixed-mode ventilation) can help maintain adequate air dilution. The threshold for ventilation requirements may vary according to the type of ventilation (e.g. recirculated air versus fresh air). In choosing a ventilation system (i.e. natural, mixed-mode, or mechanical) for health-care facilities, it is important to consider local conditions, such as building structure, climate, regulations, culture, cost, and outdoor air quality. Furthermore, any ventilation system must be monitored and maintained on a regular schedule, with adequate resources (budget and staffing) dedicated to proper maintenance. Indeed, the reason that the mechanical ventilation system in our hospital did not play an effective TB-IPC role reflects the fact that mechanical ventilation without adequate maintenance was less protective than natural ventilation. Our results align with other reports of TB infection in health facilities with faulty or no ventilation systems [20], which also require routine maintenance.

In our hospital, we note that several TB cases were detected in non-TB exposed departments, possibly due to limited TB-IPC measures taken in those hospital spaces. For example, roentgenography rooms are often poorly ventilated spaces, where droplet nuclei may remain suspended in the air for hours or even days [20]. Therefore, preventive measures such as adequate ventilation and ultraviolet-based germicidal control may also be needed in non-TB exposed environments, especially in infectious disease hospitals.

## Conclusion

In this study, UVGI, adequate ventilation, and use of a medical N95 respirator might be the risk factors of nosocomial TB transmission. In congregate settings with a high risk of TB transmission, multiple factors should be comprehensively considered to maintain TB-IPC.

## Supplementary information

**Additional file 1: Fig. S1**. TB infection rates under different TB-IPC measures for staff of Beijing Chest Hospital affiliated with Capital Medical University from 2006 to 2018. Abscissa shows the year associated with each result and the ordinate shows TB infection incidence rate. **Table S1**. The variables that have significant correlations after Spearman or Pearson analysis.

## Data Availability

Data supporting the results can be found in this paper. The datasets generated during and analyzed during the current study are available from the corresponding author on reasonable request.
