# Supplementary material for: Nosocomial tuberculosis transmission from 2006 to 2018 in Beijing Chest Hospital, China
Source: Antimicrob Resist Infect Control. 2020 Oct 24;9:165. doi: 10.1186/s13756-020-00831-5 (PMC7584851; doi:10.1186/s13756-020-00831-5)
Supplement: Supplementary file 1 — Additional file 1: Fig. S1. TB infection rates under different TB-IPC measures for staff of Beijing Chest Hospital affiliated with Capital Medical University from 2006 to 2018. Abscissa shows the year associated with each result and the ordinate shows TB infection incidence rate. Table S1. The variables that have significant correlations after Spearman or Pearson analysis. [file 13756_2020_831_MOESM1_ESM.docx]

**Supplemental figure and table**


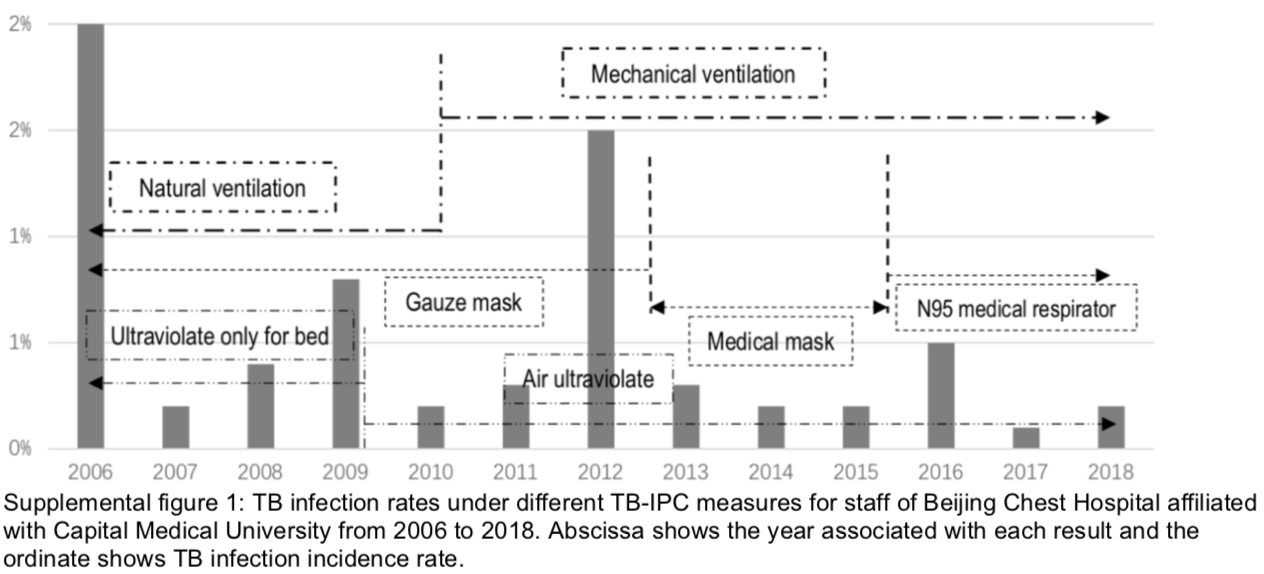


Supplemental figure 1: TB infection rates under different TB-IPC measures for staff of Beijing Chest Hospital affiliated with Capital Medical University from 2006 to 2018. Abscissa shows the year associated with each result and the ordinate shows TB infection incidence rate.

Supplement table 1: The variables that have significant correlations after Spearman or Pearson analysis.

| Variable | P value | Correlation coefficient |
| --- | --- | --- |
| Age  Duration | 0.000 | 0.886 |
| Ultraviolet  Respirator | 0.000 | 0.361 |
| Ventilation  Respirator | 0.000 | 0.408 |
| Ultraviolet  Ventilation | 0.000 | 0.539 |
